# Modeling of Electrical Conductivity for Graphene-Filled Products Assuming Interphase, Tunneling Effect, and Filler Agglomeration Optimizing Breast Cancer Biosensors

**DOI:** 10.3390/ma15186303

**Published:** 2022-09-11

**Authors:** Yasser Zare, Kyong Yop Rhee

**Affiliations:** 1 Biomaterials and Tissue Engineering Research Group, Department of Interdisciplinary Technologies, Breast Cancer Research Center, Motamed Cancer Institute, ACECR, Tehran 1125342432, Iran; 2 Department of Mechanical Engineering (BK21 Four), College of Engineering, Kyung Hee University, Yongin 17104, Korea

**Keywords:** graphene–polymer products, tunneling effect, conductivity, interphase, agglomeration

## Abstract

In this study, the percolation inception, actual filler amount, and concentration of nets are expressed using the filler size and agglomeration, interphase depth, and tunneling size. A modified form of the power-law model is recommended for the conductivity of graphene–polymer products using the mentioned characteristics. The modified model is used to plot and evaluate the conductivity at dissimilar ranges of factors. In addition, the prediction results of the model are compared with the experimented values of several samples. A low percolation inception and high-volume portion of nets that improve the conductivity of nanoparticles are achieved at a low agglomeration extent, thick interphase, large aspect ratio of the nanosheets, and large tunnels. The developed equation for percolation inception accurately predicts the results assuming tunneling and interphase parts. The innovative model predicts the conductivity for the samples, demonstrating good agreement with the experimented values. This model is appropriate to improve breast cancer biosensors, because conductivity plays a key role in sensing.

## 1. Introduction

The conductivity of nanocomposites (referred to as conductivity in this study) suddenly increases at percolation inception, because nanoparticles produce the filler network at this point [1,2]. Hence, major focus has been on the achievement of low percolation inception in nanocomposites. The aspect ratio of the filler (length per thickness/diameter) is an important parameter influencing percolation inception [3]. As such, percolation inception decreases with increasing filler aspect ratio, i.e., by big and thin nanosheets. Graphene is composed of a monolayer or a few layers of carbon with desirable mechanical, thermal, physical, and electrical properties [4,5,6,7,8,9,10,11,12,13,14,15,16,17]. The high aspect ratio of graphene nanosheets results in the establishment of conductive nets by a low filler concentration.

The size, concentration, and morphology (dispersion quality and aggregation/agglomeration) of fillers significantly impact the conductivity of polymer nanocomposites [18,19]. A higher amount of thinner and longer nanosheets increases the conductivity. Moreover, the large interfacial area of nanofillers yields the interphase part, which changes the performance of polymer nanocomposites [20,21]. A thicker interphase produces a tougher sample. Studies on the reinforcement of the interphase in the samples have found that the formation of the interphase leads to a decrease in percolation inception [2]. The positive impacts of a thick interphase on the percolation inception and conductivity have also been reported [3,22]. The conductivity of graphene products is dependent on the tunneling effect, because electrons can move over the tunnels between nearby nanoparticles based on quantum mechanics [23,24]. Hence, the percolation inception of graphene nanosheets is linked to the interphase part and tunneling effect between nanosheets, although a number of researchers have only correlated the percolation inception to filler aspect ratio [25,26].

Previous authors have applied the traditional power-law percolation model to calculate and evaluate the percolation inception and conductivity in graphene-filled samples [27,28,29]. This model applies the filler amount, percolation inception, and a factor for the estimation of conductivity, neglecting interphase deepness and tunneling dimension. Generally, there are some models for the conductivity of polymer graphene nanocomposites. However, many models disregarded the interphase or tunneling distance [30,31,32], although these parts effectively affect the percolation onset and conductivity. Actually, the former models commonly considered the amount and conductivity of graphene, as well as percolation onset in the conductivity. Moreover, recent papers considered the interphase and tunneling sections, but they neglected the agglomeration extent [24,33,34,35], which is inevitable in nanocomposites. In other words, a comprehensive model for conductivity should consider the effective terms for the graphene, interphase, and tunneling region by simple and meaningful parameters.

In this study, a modified form of the power-law model is developed for estimating the conductivity of graphene products. In our model, the percolation inception of nanoparticles is expressed using filler geometry, agglomeration extent, interphase thickness, and tunneling size. As only nanosheets belonging to the nets can improve the conductivity, the volume portion of the nets is expressed and applied in the developed model. The roles of dissimilar factors in the percolation level and concentration of nets are investigated. Furthermore, the forecasts of the model are compared with the test data. The new model is expected to help researchers study the conductivity of nanoparticles by assuming novel phenomena in nanocomposites such as the agglomeration, interphase part, and tunneling mechanism.

## 2. Theoretical Views

Hu et al. [36] expanded the power-law model for carbon nanotube (CNT)-containing samples using the aspect ratio of nanoparticles expressed as:

(1) $$\sigma =\sigma_{f}10^{0.85[\log(l/2R)-1]}{\left(\phi_{f}-\phi_{p}\right)}^{b}$$

where *σ_f_*, *l*, *R*, $\phi_{f}$, and $\phi_{p}$ denote the conduction, length, radius, volume portion, and percolation inception of the filler, respectively, and *b* is an exponent. Hu et al. reported that the model correctly forecast the conductivity, although the filler agglomeration, interphase part, and tunneling effect were not considered. In this study, Equation (1) is advanced for determining the conductivity of graphene products.

The percolation inception in the materials consisting of haphazardly oriented graphite is given [37] below:

(2) $$\phi_{p}=\frac{27\pi D^{2}t}{4{\left(D+d\right)}^{3}}$$

where *t* and *D* are the thickness and diameter of the nanosheets, respectively, and *d* is the tunneling size between the adjacent sheets. When *D* >> *d*, the latter equation can be simplified as follows:

(3) $$\phi_{p}=\frac{27\pi t}{4D}$$

The interphase part shifts the percolation inception to smaller filler portions. The tunneling spaces produce the conductive nets between neighboring nanosheets with small distances separating them. From this analysis, it can be suggested that the tunneling and interphase parts decrease the percolation inception as shown:

(4) $$\phi_{p}=\frac{27\pi t}{4D+2(Dt_{i}+Dd)}$$

where *t_i_* denotes the interphase depth.

The aspect ratio is *α* = *D*/*t*, so the latter equation is restructured into:

(5) $$\phi_{p}=\frac{27\pi }{(4+2t_{i}+2d)\alpha }$$

Due to the high superficial energy of nanoparticles and van der Waals attraction between nanosheets, the agglomeration of nanosheets occurs in the system [38]. The nanosheets assume a sphere-like structure in the nanocomposite, which seriously reduces their aspect ratio [36].

The effect of agglomeration on the aspect ratio is expressed as:

(6) $$\alpha_{g}=\frac{\alpha }{g}$$

where *g* shows the extent of agglomeration. When *g* = 1, well-dispersed nanosheets are displayed in the nanocomposite (no agglomeration); a higher *g* value due to a stronger agglomeration reduces the aspect ratio. *g* is determined by the size of agglomerated graphene. For example, when the thickness of the nanosheets increases two times due to agglomeration, *g* = 2. Actually, *g* depends on the agglomeration size. The value of *g* can be determined using morphological images.

Assuming the agglomeration of nanoparticles due to a reduced aspect ratio, the percolation inception can be expressed by substituting Equation (6) into Equation (5) as shown:

(7) $$\phi_{p}=\frac{27\pi g}{(4+2t_{i}+2d)\alpha }$$

This equation can be utilized to calculate the percolation inception in nanocomposites. Low percolation inception is realized assuming the tunneling and interphase part, while agglomeration increases it.

The interphase part increases the actual concentration of nanoparticles in nanocomposites. The volume portion of the interphase part in polymer graphene nanocomposites [39] is expressed as:

(8) $$\phi_{i}=\phi_{f}(\frac{2t_{i}}{t})$$

The actual volume portion of graphene in nanocomposites contains the total portions of nanoparticles and the interphase as:

(9) $$\phi_{eff}=\phi_{f}+\phi_{i}=\phi_{f}(1+\frac{2t_{i}}{t})$$

Therefore, the thicknesses of nanosheets and the interphase control the actual filler concentration in nanocomposites.

The portion of nanoparticles contributing to the conductive nets [40] is expressed by percolation inception and filler concentration as:

(10) $$f=\frac{\phi_{f}^{1/3}-\phi_{p}^{1/3}}{1-\phi_{p}^{1/3}}$$

where *f* can be developed by the interphase part, tunneling effect, and agglomeration level, when the actual filler concentration and percolation inception from Equations (7) and (9) are considered as:

(11) $$f=\frac{\phi_{eff}^{1/3}-\phi_{p}^{1/3}}{1-\phi_{p}^{1/3}}$$

Nets handle the conductivity because of their ability to transfer charge, while the detached particles have no effect on the conductivity. Consequently, estimating the volume portion of nets is important.

The volume portion of nanosheets precipitating in the nets can be calculated as follows:

(12) $$\phi_{N}^{}=f\phi_{f}^{}$$

when *f* from Equation (11) is substituted into the above equation and the actual concentration of nanoparticles is considered, $\phi_{N}^{}$ can be calculated using:

(13) $$\phi_{N}^{}=\frac{\phi_{eff}^{1/3}-\phi_{p}^{1/3}}{1-\phi_{p}^{1/3}}\phi_{eff}^{}$$

which relates to the sum and size of graphene, interphase depth, tunneling size, and agglomeration extent.

Equation (1) can now be developed for the conductivity of graphene products by interphase depth, tunneling size, filler agglomeration, and nets as follows:

(14) $$\sigma =\sigma_{f}10^{0.85[\log(\alpha_{g})-1]}{\left(\phi_{N}-\phi_{p}\right)}^{b}$$

where *α_g_*, $\phi_{N}^{}$, and $\phi_{p}^{}$ are from Equations (6), (7), and (13), respectively. This model demonstrates the impacts of several factors such as the interphase, tunneling effect, agglomeration, and conductive nets on the conductivity.

## 3. Results and Discussion

### 3.1. Percolation Inception

The stimuli of dissimilar factors on the percolation inception were studied using the developed equation (Equation (7)). Each contour plot shows the impacts of two factors on the percolation level at *D* = 2 μm, *t* = 2 nm, *t_i_* = 5 nm, *d* = 5 nm, and *g* = 1.5.

**Figure 1:**
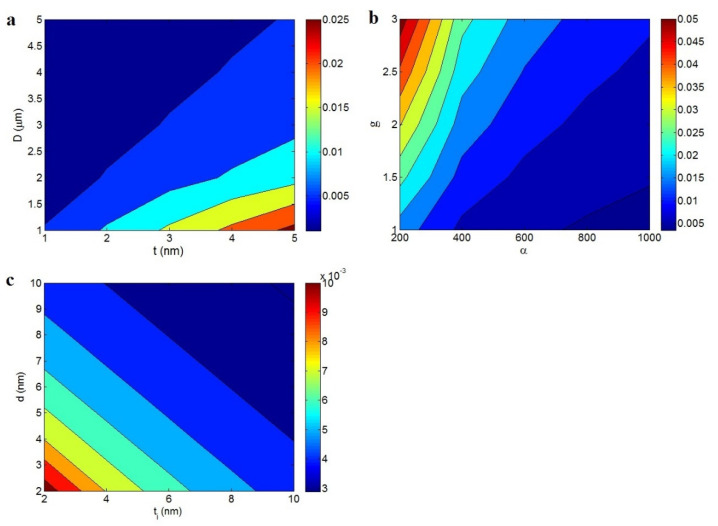
Stimuli of (**a**) *t* and *D*, (**b**) *α* and *g*, and (**c**) *t_i_* and *d* on the percolation inception based on Equation (7).

Figure 1a illustrates the impressions of *t* and *D* on the percolation inception. Low *t* and high *D* produced a small percolation level, and the highest percolation inception was perceived at the highest value of *t* and the smallest *D*. Accordingly, thin and large nanosheets, which participate in conductive nets because of the large part they cover in nanocomposites, obtained a desirable percolation inception. In other words, strong interactions and contacts among thin and large nanosheets occurred in conductive nets.

Figure 1b shows the percolation inception at different levels of *α* and *g*. The lowest percolation level of approximately 0.005 was obtained at *α* = 1000 and *g* = 1, whereas the percolation level undesirably increased to 0.05 at *α* = 200 and *g* = 3. Accordingly, a high *α* and low *g* yielded a low percolation inception. In other words, the smallest percolation inception was realized by the highest aspect ratio and the lowest agglomeration. The percolation inception is inversely proportional to the aspect ratio, because a high aspect ratio by thin and large sheets decreases the percolation level. A high level of agglomeration lowers the number and aspect ratio in the nanocomposites, weakening the networking in the nanocomposites. A low level of agglomeration results in a desirable dispersion of the high-aspect-ratio filler in the samples, causing the formation of nets by the small concentration of nanosheets. As a result, the percolation inception is directly linked to the agglomeration level.

Figure 1c illustrates the stimuli of *t_i_* and *d* on the percolation inception. These parameters inversely affected the percolation inception, and the lowest percolation level of 0.003 was obtained at *t_i_* = *d* > 7 nm. A poor percolation inception was obtained from a thick interphase and large tunneling size. A thick interphase surrounding the nanosheets reduces the separation distance and produces conductive nets from a small amount of nanosheets. However, a thin interphase does not affect the percolation level because it does not change the distance between nanosheets. In contrast, the conductive nets forming the nanosheets are separated by the tunneling size. Therefore, a large tunneling size can involve a significant number of nanosheets in the percolated nets, creating a low percolation inception. This demonstrates that obtaining a low percolation inception from a thick interphase and large tunneling size is achievable.

### 3.2. Volume Portion of Nets

**Figure 2:**
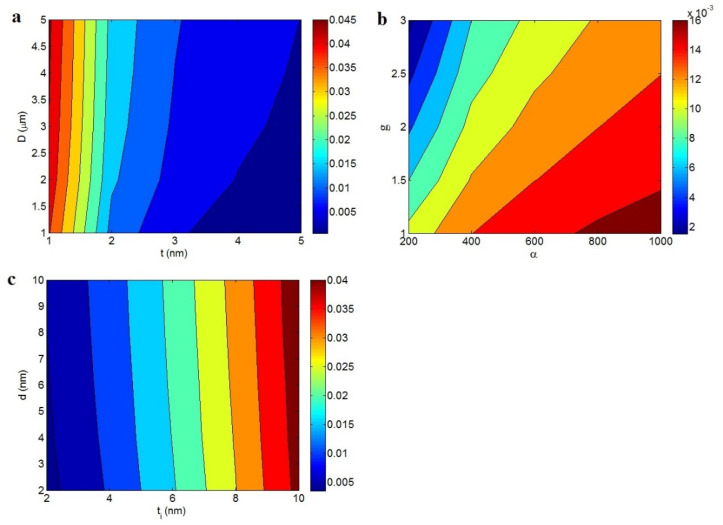
Altered points of (**a**) *t* and *D*, (**b**) *α* and *g*, and (**c**) *t_i_* and *d* assuming the interphase part, tunneling size, and filler agglomeration according to Equation (13).

The impacts of various parameters on the volume portion of percolated nanosheets (Equation (13)) are illustrated in Figure 2. The average ranking of factors in the predictions was deliberated at *t* = 2 nm, $\phi_{f}$ = 0.01, *t_i_* = 5 nm, *d* = 5 nm, *D* = 2 μm, and *g* = 1.5.

Figure 2a illustrates the impressions of *t* and *D* on $\phi_{N}$. The maximum value of $\phi_{N}$ = 0.45 was achieved at *t* = 1 nm and *D* = 5 μm, although $\phi_{N}$ decreased to 0 at *t* > 4 nm and *D* < 2.5 μm. Thin and large nanosheets yielded a high portion of percolated nets in the samples, which resulted in the reduction of the percolation level. Thin nanosheets produced a desirable interphase part in the nanocomposites based on Equation (8). Therefore, it is logical to obtain a high portion of percolated nanosheets by thin and large nanosheets, because they make big nets including the interphase part and nanosheets in the nanocomposites.

Figure 2b depicts the effects of *α* and *g* on $\phi_{N}$. The best outputs were obtained at maximum *α* and minimum *g*; however, $\phi_{N}$ significantly decreased at low *α* and high *g* values. A more desirable $\phi_{N}$ was achieved for a higher aspect ratio and less agglomeration. The favorable roles of the aspect ratio in the percolation level and interphase part are predictable due to a large interphase area yielded by a high aspect ratio, which decreases the percolation level and promotes filler concentration in the samples. Similarly, a higher aspect ratio leads to a higher $\phi_{N}$ in nanocomposites. In contrast, a high *g* lowers the aspect ratio and superficial part of the nanofiller, which negatively impacts the percolation level and the effectiveness of the nanoparticles. A high *g* limits the interphase part in the nanocomposite, which destructively alters the percolation inception and actual filler concentration. The developed equation suggests a high $\phi_{N}$ from a high aspect ratio and low agglomeration.

The influences of tunneling size and interphase depth on the volume quota of percolated nanosheets are shown in Figure 2c. The highest $\phi_{N}$ was obtained from the thickest interphase, whereas the thinnest interphase and the shortest tunneling size produced the lowest $\phi_{N}$. It can be concluded that the interphase depth dominantly affects $\phi_{N}$ due to the thickness of the interphase layer around nanoparticles controlling the percolation inception and actual filler concentration, whereas the tunneling size only affects the percolation level. A profuse interphase lowers the percolation level and causes a high actual filler portion resulting from the large interphase area produced in the nanocomposites. However, a large tunneling size decreases the percolation inception and has no effect on the effectiveness of nanoparticles. A large $\phi_{N}$ is expected when using a deep interphase, whereas a thin interphase and short tunneling size negatively affect $\phi_{N}$.

### 3.3. Electrical Conductivity

#### 3.3.1. Parameter Effects

The conductivity of polymer graphene nanocomposites was expressed by the new model in Equation (14) at different ranges of factors when *b* = 6.

**Figure 3:**
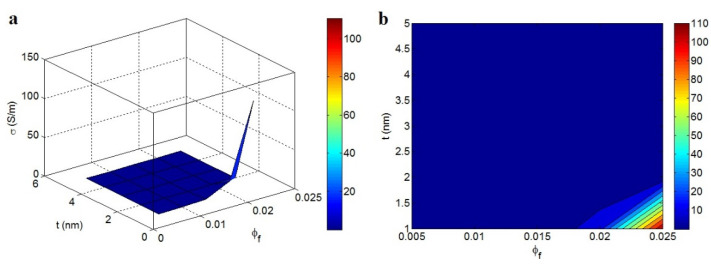
Conductivity when $\phi_{f}$ and *t* are substituted into Equation (14): (**a**) three-dimensional (3D) and (**b**) two-dimensional (2D) illustrations.

Figure 3 illustrates the effects of $\phi_{f}$ and *t* on the conductivity. The conductivity increased to 110 S/m at $\phi_{f}$ = 0.025 and *t* = 1 nm, and an insulated product was perceived at $\phi_{f}$ < 0.018 or *t* > 2 nm. A high filler amount and thin nanosheets significantly increased the conductivity, whereas a low filler amount and dense nanosheets did not increase the conductivity.

A low filler portion in a nanocomposite does not alter the percolation value required to increase the conductivity. In contrast, a high portion of graphene yields large and compact nets in the nanocomposite capable of transferring charge and increasing the conductivity. Thin nanosheets positively affect the percolation level, interphase part, and net efficiency, as they reduce the percolation inception, thicken the interphase layer, and increase the size and compactness of nets.

**Figure 4:**
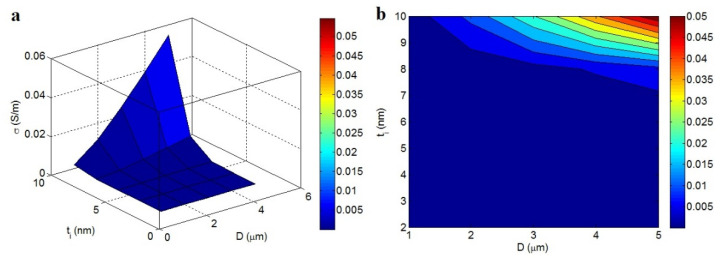
Effects of *D* and *t_i_* on the predicted conduction using Equation (14): (**a**) 3D and (**b**) 2D pictures.

Figure 4 portrays the variations of conductivity at different collections of *D* and *t_i_*. The highest conductivity of 0.05 S/m was obtained at *D* = 5 μm and *t_i_* = 10 nm, whereas a very poor conductivity was obtained at *D* < 1.4 μm and *t_i_* < 7 nm. Thus, the diameter of the nanosheets and interphase depth govern conductivity, and the maximum conductivity was reached for the largest nanosheets and thickest interphase.

Large nanosheets enhance the aspect ratio, lower the percolation level, and develop the portion of nanosheets in the nets, thereby significantly increasing the conductivity due to the linkage with net properties [41,42]. Large nanosheets produce big nets, which transport charge and create strong conductivity. Moreover, a plentiful interphase supports the efficiency of nanoparticles, as the interphase part can enlarge the nets. A dense interphase moves the percolation level to smaller filler portions and increases the actual filler portion. These desirable levels produce favorable nets, which raise the charge conveyance in the nanocomposite. Better conductivity is observed from a thicker interphase layer as compared to a thin interphase, which has no effect on the general performance of nanocomposites. Literature studies have discussed the positive impacts of the interphase on the percolation inception and conductivity of polymer CNT nanocomposites [40].

**Figure 5:**
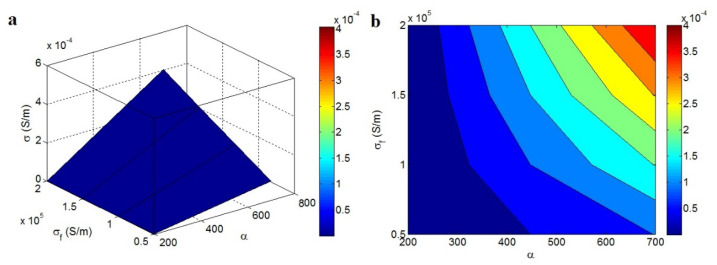
Predicted conductivity using *α* and *σ_f_*: (**a**) 3D and (**b**) contour pictures.

The dependency of conductivity on *α* and *σ_f_* is illustrated in Figure 5 ($\phi_{f}$ = 0.015). The best value of conductivity was achieved when the heights of *α* and *σ_f_* were at the maximum. This observation demonstrates that conductivity is controlled by the aspect ratio and conduction of graphene sheets. A high aspect ratio decreases the percolation level and increases the share of nanosheets in the nets, thereby governing the conductivity. Graphene is the only conductive nanomaterial in nanocomposites, and its conduction level affects the conductivity.

Previous studies discussed the direct influences of t, the aspect ratio, and the conduction of nanoparticles on the conductivity [43]. Therefore, increased conductivity can be achieved from high levels of the aspect ratio and filler condition.

**Figure 6:**
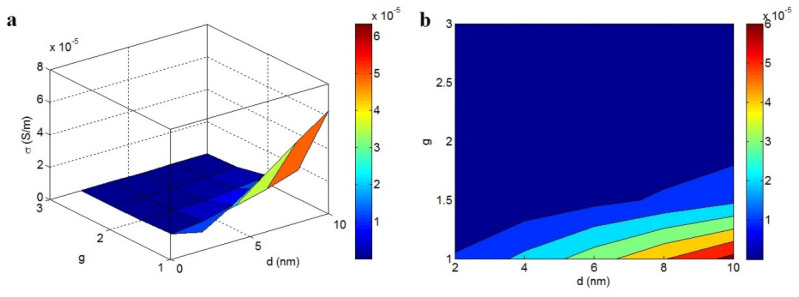
Disparities of conductivity at different ranks of *d* and *g*: (**a**) 3D and (**b**) 2D configurations.

Figure 6 shows the effects of *d* and *g* on conductivity. The highest conductivity of 6 × 10^−5^ S/m was achieved at *d* = 10 nm and *g* = 1. An insulated nanocomposite was achieved at high g levels. The diagram shows that a large tunneling size and poor agglomeration increased the conductivity, whereas the agglomeration of nanoparticles seriously weakened it.

A large tunneling size can allow the participation of the far nanosheets in the conductive nets. Large nets are constructed by large tunnels, which improve the conductivity; however, the maximum level of the tunneling size reported in previous studies was 10 nm [44]. This means that tunneling sizes greater than 10 nm are ineffective at producing conductive nanocomposites. Nanocomposites are conductive when electron transfer between the nanosheets occurs. The current model assumes the tunneling size using percolation inception (Equation (7)) and shows a direct dependency of conductivity on the tunneling size; in contrast, other models displayed an opposite link between conductivity and tunneling size [45,46]. The models observed the different influences of the tunneling size on the conductivity due to a large tunnel, causing simultaneously quick percolation and weak electron transport.

The agglomeration of nanosheets reduces the superficial area and aspect ratio of the nanofiller and induces a large percolation inception and small interphase part. The poor conductivity obtained from higher agglomeration is a result of the small conductive nets produced, which do not transfer charge. The undesirable effect of agglomeration on the rigidity of nanocomposites was also mentioned in previous studies [47,48]. It can be concluded that agglomeration negatively governs conductivity. Furthermore, these factors negligibly affect the conductivity from 0 to 6 × 10^−5^ S/m.

**Figure 7:**
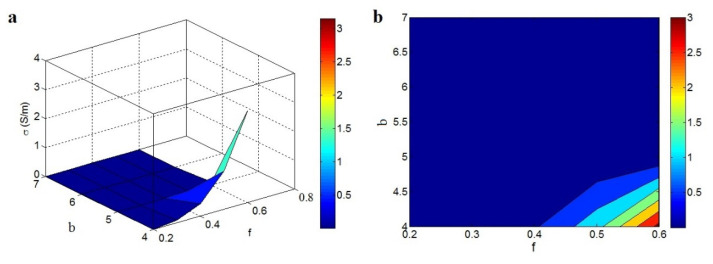
Correspondences of conductivity to *f* and *b*: (**a**) 3D and (**b**) 2D designs.

The effects of *f* and *b* on the conductivity are illustrated in Figure 7. The uppermost conductivity of 3 S/m was attained when *f* = 0.6 and *b* = 4, whereas the conductivity was almost 0 at *f* < 0.4 or *b* > 4.8. An insulated sample was produced from the low percentage of nanosheets in the nets and the high level of *b*, whereas a high portion of nanoparticles in the nets and low *b* produced better conductivity.

The effect of *f* on the conductivity is reasonable, as large nets can efficiently transfer charge that establishes desirable conductivity. Additionally, a higher level of *b* suggests poor conductivity due to the weakened effect of the net volume portion in the conductivity. Recent studies on the original percolation models reported a similar influence of the b exponent on the conductivity, that is poor conductivity was observed when the b exponent level was high [27,28,29]. The current model shows the influences of *f* and *b* on the conductivity.

#### 3.3.2. Examination of Developed Model by Experimental Data

**Figure 8:**
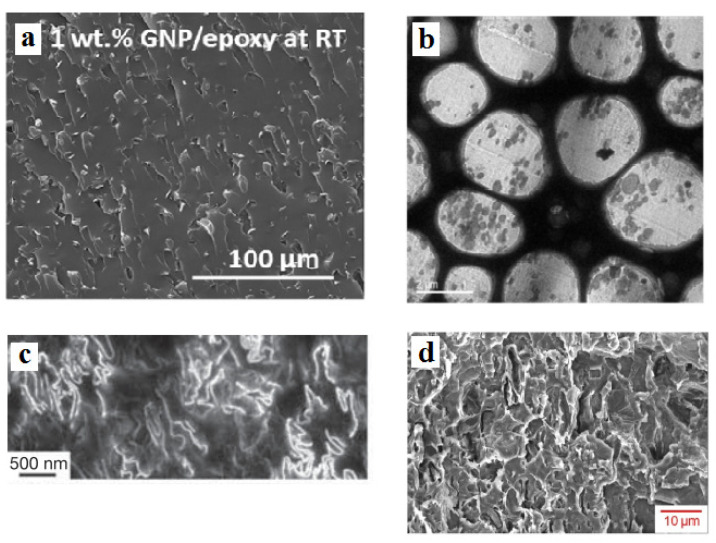
Morphological images of (**a**) epoxy [49], (**b**) ABS [50], (**c**) PS [51], and (**d**) PVDF [28] graphene systems.

Several graphene–polymer nanocomposites comprising epoxy [49], acrylonitrile butadiene styrene (ABS) [50], polystyrene (PS) [51], and poly(vinylidene fluoride) (PVDF) [28] were selected from previous articles (Table 1). All details of the experimental processes and synthesis of the nanocomposites are available in the references. We only indicate the processing technique of the samples, the graphene dimensions, and the percolation onset from the original references in Table 1. Moreover, morphological images of these nanocomposites are depicted in Figure 8. Good dispersion of graphene nanosheets and the formation of networks after percolation onset are evident in the morphological images. Undoubtedly, the conductivity of samples relates to the dispersion quality and size of the nanosheets, and the morphological pictures are useful to analyze the structure and dimensions of the nanosheets. Furthermore, conductivity determinations were detailed in the original references.

First, the experimental percolation onset was substituted into Equation (7) to determine the average values of interphase deepness and tunneling size, as presented in Table 1. A thick interphase and large tunnels were observed in the PS sample, due to the lowest percolation level. It can be concluded that the developed equation correctly predicts the percolation inception by the tunneling and interphase parts. Using the developed model (*g* = 1 and *σ_f_* = 10^5^ S/m), the conductivity of the reported samples was estimated.

**Figure 9:**
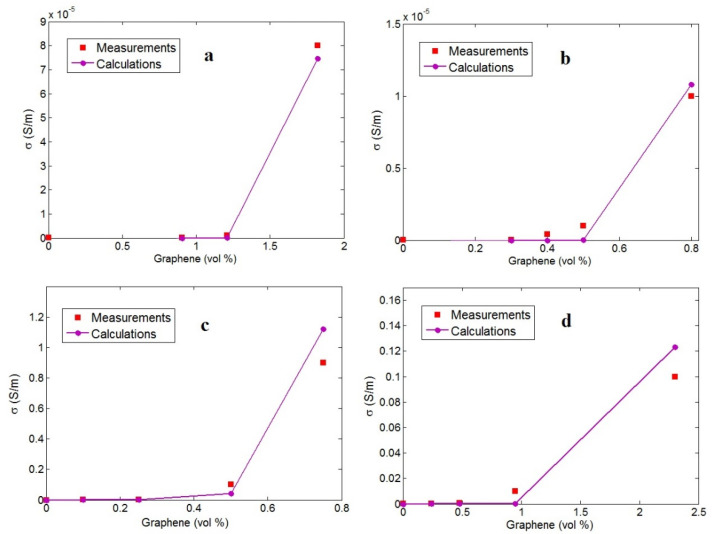
Experimental measurements and predictions using Equation (14) at different filler amounts for (**a**) epoxy [49], (**b**) ABS [50], (**c**) PS [51], and (**d**) PVDF [28] graphene systems.

Figure 9 shows the experimented and predicted conductivity values estimated for the examples. The developed model is able to predict the percolation-like behavior of conductivity based on the test results. Consequently, the developed model predicts the conductivity considering the stimuli of the interphase part, tunnels, agglomeration, and nets. The values of *b* exponent were also calculated, as presented in Table 1. The highest *b* value of 7.3 was observed in the ABS/graphene nanocomposite due to the poor conductivity levels. Other samples displayed different ranks for the conductivity and *b* exponent. Since the calculated parameters for the interphases, tunnels, and *b* are reasonable and meaningful, the developed methodology and its outputs are validated.

## 4. Conclusions

A modified power-law equation was proposed to visualize the conductivity of a graphene–polymer system using various parameters related to the interphase part, tunneling effect, and agglomeration. The conductivity was evaluated by the effects of various parameters and experimented records of numerous samples. The desired levels of the percolation inception, volume portion of percolated nanosheets, and conductivity were obtained by thinner and larger nanosheets, a poorer agglomeration, a thicker interphase, and a larger tunneling size. High conductivity was obtained from the high concentration and high conduction of graphene and a low *b* exponent. The large variations of conductivity were observed when the concentration and thickness of the graphene nanosheets changed, whereas several parameters such as the tunneling size, agglomeration level, aspect ratio, and filler conduction negligibly affected the conductivity. The model predicted the percolation-like behavior of conductivity for the samples, and the results showed good agreement with the experimented control levels. The new model is appropriate to improve the performance of breast cancer biosensors, since conductivity plays an important role in the detection of cancer cells.

## Figures and Tables

**Table 1:** Information of graphene–polymer samples.

Matrix [Ref.]	Processing Technique	*D* (μm)	*t* (nm)	$\phi_{p}^{}$	*t_i_* (nm)	*d* (nm)	*b*
Epoxy [49]	In situ dispersion	2	2	0.0050	2	4	5.2
ABS [50]	Coagulation method	4	1	0.0013	3	3	7.3
PS [51]	Solution mixing	2	1	0.0010	8	8	5.5
PVDF [28]	Solution mixing	2	1	0.0030	2	3	5.4

## Data Availability

The raw data required to reproduce these findings are available upon request from the corresponding author.

## References

[1] Wang H., Niu B., Chen M., Hao L., Cao X., Jiang S. (2017). Facile layer-by-layer assembly to construct methoxybenzene group functionalized graphene/poly (ethylene-co-vinyl alcohol) barrier films under parallel electric field. Mater. Des..

[2] Kazemi F., Mohammadpour Z., Naghib S.M., Zare Y., Rhee K.Y. (2021). Percolation onset and electrical conductivity for a multiphase system containing carbon nanotubes and nanoclay. J. Mater. Res. Technol..

[3] Zare Y., Rhee K.Y. (2017). Accounting the reinforcing efficiency and percolating role of interphase regions in the tensile modulus of polymer/CNT nanocomposites. Eur. Polym. J..

[4] Kim S.-H., Zhang Y., Lee J.-H., Lee S.-Y., Kim Y.-H., Rhee K.Y., Park S.-J. (2021). A study on interfacial behaviors of epoxy/graphene oxide derived from pitch-based graphite fibers. Nanotechnol. Rev..

[5] Wei D., Liu X., Lv S., Liu L., Wu L., Li Z., Hou Y. (2021). Fabrication, Structure, Performance, and Application of Graphene-Based Composite Aerogel. Materials.

[6] Storti E., Fruhstorfer J., Luchini B., Jiříčková A., Jankovský O., Aneziris C.G. (2021). Graphene-Reinforced Carbon-Bonded Coarse-Grained Refractories. Materials.

[7] Chungyampin S., Niamlang S. (2021). The Soft and High Actuation Response of Graphene Oxide/Gelatin Soft Gel. Materials.

[8] Alborzi M.S., Rajabpour A. (2021). Thermal transport in van der Waals graphene/boron-nitride structure: A molecular dynamics study. Eur. Phys. J. Plus.

[9] Bahrami S., Baheiraei N., Shahrezaee M. (2021). Biomimetic reduced graphene oxide coated collagen scaffold for in situ bone regeneration. Sci. Rep..

[10] Sieradzka M., Fabia J., Biniaś D., Graczyk T., Fryczkowski R. (2021). High-Impact Polystyrene Reinforced with Reduced Graphene Oxide as a Filament for Fused Filament Fabrication 3D Printing. Materials.

[11] Zafeiropoulou K., Kostagiannakopoulou C., Geitona A., Tsilimigkra X., Sotiriadis G., Kostopoulos V. (2021). On the Multi-Functional Behavior of Graphene-Based Nano-Reinforced Polymers. Materials.

[12] Zeranska-Chudek K., Wróblewska A., Kowalczyk S., Plichta A., Zdrojek M. (2021). Graphene infused ecological polymer composites for electromagnetic interference shielding and heat management applications. Materials.

[13] Keshvardoostchokami M., Piri F., Jafarian V., Zamani A. (2020). Fabrication and antibacterial properties of silver/graphite oxide/chitosan and silver/reduced graphene oxide/chitosan nanocomposites. JOM.

[14] Pagnola M., Morales F., Tancredi P., Socolovsky L. (2021). Radial Distribution Function Analysis and Molecular Simulation of Graphene Nanoplatelets Obtained by Mechanical Ball Milling. JOM.

[15] Alimohammadian M., Sohrabi B. (2020). Manipulating electronic structure of graphene for producing ferromagnetic graphene particles by Leidenfrost effect-based method. Sci. Rep..

[16] Naghib S.M., Behzad F., Rahmanian M., Zare Y., Rhee K.Y. (2020). A highly sensitive biosensor based on methacrylated graphene oxide-grafted polyaniline for ascorbic acid determination. Nanotechnol. Rev..

[17] Sagadevan S., Shahid M.M., Yiqiang Z., Oh W.-C., Soga T., Lett J.A., Alshahateet S.F., Fatimah I., Waqar A., Paiman S. (2021). Functionalized graphene-based nanocomposites for smart optoelectronic applications. Nanotechnol. Rev..

[18] Niklaus M., Shea H. (2011). Electrical conductivity and Young’s modulus of flexible nanocomposites made by metal-ion implantation of polydimethylsiloxane: The relationship between nanostructure and macroscopic properties. Acta Mater..

[19] Clingerman M.L., King J.A., Schulz K.H., Meyers J.D. (2002). Evaluation of electrical conductivity models for conductive polymer composites. J. Appl. Polym. Sci..

[20] Zare Y., Rhim S.S., Rhee K.Y. (2021). Development of Jang–Yin model for effectual conductivity of nanocomposite systems by simple equations for the resistances of carbon nanotubes, interphase and tunneling section. Eur. Phys. J. Plus.

[21] Baek K., Shin H., Cho M. (2021). Multiscale modeling of mechanical behaviors of Nano-SiC/epoxy nanocomposites with modified interphase model: Effect of nanoparticle clustering. Compos. Sci. Technol..

[22] Shin H., Yang S., Choi J., Chang S., Cho M. (2015). Effect of interphase percolation on mechanical behavior of nanoparticle-reinforced polymer nanocomposite with filler agglomeration: A multiscale approach. Chem. Phys. Lett..

[23] Fang C., Zhang J., Chen X., Weng G.J. (2019). A Monte Carlo model with equipotential approximation and tunneling resistance for the electrical conductivity of carbon nanotube polymer composites. Carbon.

[24] Payandehpeyman J., Mazaheri M., Khamehchi M. (2020). Prediction of electrical conductivity of polymer-graphene nanocomposites by developing an analytical model considering interphase, tunneling and geometry effects. Compos. Commun..

[25] Martins J.N., Bassani T.S., Barra G.M., Oliveira R.V. (2011). Electrical and rheological percolation in poly (vinylidene fluoride)/multi-walled carbon nanotube nanocomposites. Polym. Int..

[26] McClory C., McNally T., Baxendale M., Pötschke P., Blau W., Ruether M. (2010). Electrical and rheological percolation of PMMA/MWCNT nanocomposites as a function of CNT geometry and functionality. Eur. Polym. J..

[27] Lan Y., Liu H., Cao X., Zhao S., Dai K., Yan X., Zheng G., Liu C., Shen C., Guo Z. (2016). Electrically conductive thermoplastic polyurethane/polypropylene nanocomposites with selectively distributed graphene. Polymer.

[28] He L., Tjong S.C. (2013). Low percolation threshold of graphene/polymer composites prepared by solvothermal reduction of graphene oxide in the polymer solution. Nanoscale Res. Lett..

[29] Tu Z., Wang J., Yu C., Xiao H., Jiang T., Yang Y., Shi D., Mai Y.-W., Li R.K. (2016). A facile approach for preparation of polystyrene/graphene nanocomposites with ultra-low percolation threshold through an electrostatic assembly process. Compos. Sci. Technol..

[30] Manta A., Gresil M., Soutis C. (2017). Predictive model of graphene based polymer nanocomposites: Electrical performance. Appl. Compos. Mater..

[31] Mutlay İ., Tudoran L.B. (2014). Percolation behavior of electrically conductive graphene nanoplatelets/polymer nanocomposites: Theory and experiment. Fuller. Nanotub. Carbon Nanostruct..

[32] Yoonessi M., Gaier J.R., Sahimi M., Daulton T.L., Kaner R.B., Meador M.A. (2017). Fabrication of graphene–polyimide nanocomposites with superior electrical conductivity. ACS Appl. Mater. Interfaces.

[33] Lu X., Yvonnet J., Detrez F., Bai J. (2017). Multiscale modeling of nonlinear electric conductivity in graphene-reinforced nanocomposites taking into account tunnelling effect. J. Comput. Phys..

[34] Mazaheri M., Payandehpeyman J., Khamehchi M. (2020). A developed theoretical model for effective electrical conductivity and percolation behavior of polymer-graphene nanocomposites with various exfoliated filleted nanoplatelets. Carbon.

[35] Zare Y., Rhee K.Y. (2022). Effect of contact resistance on the electrical conductivity of polymer graphene nanocomposites to optimize the biosensors detecting breast cancer cells. Sci. Rep..

[36] Hu N., Masuda Z., Yan C., Yamamoto G., Fukunaga H., Hashida T. (2008). The electrical properties of polymer nanocomposites with carbon nanotube fillers. Nanotechnology.

[37] Li J., Kim J.-K. (2007). Percolation threshold of conducting polymer composites containing 3D randomly distributed graphite nanoplatelets. Compos. Sci. Technol..

[38] Alian A., El-Borgi S., Meguid S. (2016). Multiscale modeling of the effect of waviness and agglomeration of CNTs on the elastic properties of nanocomposites. Comput. Mater. Sci..

[39] Yanovsky Y.G., Kozlov G., Karnet Y.N. (2013). Fractal description of significant nano-effects in polymer composites with nanosized fillers. Aggregation, phase interaction, and reinforcement. Phys. Mesomech..

[40] Feng C., Jiang L. (2013). Micromechanics modeling of the electrical conductivity of carbon nanotube (CNT)–polymer nanocomposites. Compos. Part A Appl. Sci. Manuf..

[41] Thostenson E.T., Chou T.W. (2006). Carbon nanotube networks: Sensing of distributed strain and damage for life prediction and self healing. Adv. Mater..

[42] Yang J., Zhang Y., Wang Z., Chen P. (2014). Influences of high aspect ratio carbon nanotube network on normal stress difference measurements and extrusion behaviors for isotactic polypropylene nanocomposite melts. RSC Adv..

[43] Taherian R. (2016). Experimental and analytical model for the electrical conductivity of polymer-based nanocomposites. Compos. Sci. Technol..

[44] Li J., Ma P.C., Chow W.S., To C.K., Tang B.Z., Kim J.K. (2007). Correlations between percolation threshold, dispersion state, and aspect ratio of carbon nanotubes. Adv. Funct. Mater..

[45] Maiti S., Suin S., Shrivastava N.K., Khatua B. (2013). Low percolation threshold in polycarbonate/multiwalled carbon nanotubes nanocomposites through melt blending with poly (butylene terephthalate). J. Appl. Polym. Sci..

[46] Ambrosetti G., Grimaldi C., Balberg I., Maeder T., Danani A., Ryser P. (2010). Solution of the tunneling-percolation problem in the nanocomposite regime. Phys. Rev. B.

[47] Zare Y., Rhee K.Y. (2019). A simulation work for the influences of aggregation/agglomeration of clay layers on the tensile properties of nanocomposites. JOM.

[48] Zare Y. (2016). Modeling the yield strength of polymer nanocomposites based upon nanoparticle agglomeration and polymer–filler interphase. J. Colloid Interface Sci..

[49] Li Y., Zhang H., Porwal H., Huang Z., Bilotti E., Peijs T. (2017). Mechanical, electrical and thermal properties of in-situ exfoliated graphene/epoxy nanocomposites. Compos. Part A Appl. Sci. Manuf..

[50] Gao C., Zhang S., Wang F., Wen B., Han C., Ding Y., Yang M. (2014). Graphene networks with low percolation threshold in ABS nanocomposites: Selective localization and electrical and rheological properties. ACS Appl. Mater. Interfaces.

[51] Stankovich S., Dikin D.A., Dommett G.H., Kohlhaas K.M., Zimney E.J., Stach E.A., Piner R.D., Nguyen S.T., Ruoff R.S. (2006). Graphene-based composite materials. Nature.

